# Indium Tin Oxide Substrates Enable Coating-Free SEM Imaging and Simplified Preparation of Purified Fibrinogen Clots

**DOI:** 10.1093/mam/ozag116

**Published:** 2026-11-11

**Authors:** Can Cai, Caela Flake, Arezoo Nameny, Nathan E Hudson, Brittany E Bannish, Martin Guthold

**Affiliations:** Department of Physics, Wake Forest University, 2090 Eure Drive, Winston-Salem, NC 27109, USA; Department of Physics, Wake Forest University, 2090 Eure Drive, Winston-Salem, NC 27109, USA; Department of Physics, Wake Forest University, 2090 Eure Drive, Winston-Salem, NC 27109, USA; Department of Physics, East Carolina University, 1000 E. 5th Street, Greenville, NC 27885, USA; Department of Mathematics and Statistics, University of Central Oklahoma, 100 N. University Drive, Edmond, OK 73034, USA; Department of Physics, Wake Forest University, 2090 Eure Drive, Winston-Salem, NC 27109, USA

**Keywords:** biopolymer network structure, fibrin network structure, fibrinogen, thrombin

## Abstract

Scanning electron microscopy (SEM) of fibrin fibers typically requires time-consuming preparation and conductive sputter coating, which can alter nanoscale structural measurements. Additionally, protocols for purified fibrinogen clots often include unnecessary steps adapted from plasma clot methods. We aimed to evaluate indium tin oxide (ITO) as a flat, conductive SEM substrate, investigate sputter coating effects on fiber diameter, and simplify purified fibrinogen clot preparation. Platelet-poor plasma and purified fibrinogen clots were formed on ITO substrates and imaged via SEM after 0, 45, or 90 s of sputter coating to quantify diameter variations. For purified clots, a simplified protocol—forming and imaging clots directly on ITO—was compared against a standardized workflow involving clot transfer from microtube lids to carbon tape. Sputter coating duration significantly affected fiber measurements, with longer coating times increasing apparent diameters. Plasma and purified clots showed distinct responses. Crucially, the simplified ITO protocol produced fiber diameter measurements that did not differ significantly from the standardized workflow under identical coating conditions. ITO is an effective conductive substrate that simplifies purified fibrinogen clot preparation by eliminating washing and transfer steps. It yields comparable diameter measurements to standard methods, offering an efficient platform for analyzing fibrin structures.

## Introduction

Blood clot formation is essential for hemostasis; however, abnormal clot formation and impaired clot breakdown are associated with numerous diseases, including myocardial infarction, ischemic stroke, venous thromboembolism, pulmonary embolism, diabetes, and cardiovascular disease (Undas & Ariens, 2011; Ząbczyk & Undas, 2017). Cardiovascular diseases remain the leading cause of death worldwide and account for approximately one-third of all global deaths. Because fibrin forms the primary structural scaffold of blood clots, understanding how fibrin structure changes under different physiological and pathological conditions is important for understanding clot function and disease progression (Weisel & Litvinov, 2013).

Over the past several decades, electron microscopy has become one of the most important tools for studying blood clot ultrastructure. It has been used to investigate fibrin polymerization, protein–protein interactions, clot architecture, and structure–function relationships within fibrin networks (Medved, et al., 1990; Chernysh, et al., 2011; Cai, et al., 2026*b*). Numerous studies have shown that fibrin morphology is associated with disease state, clot mechanical properties, permeability, and susceptibility to fibrinolysis. Structural characteristics such as fiber diameter, fiber length, branching, network density, and pore size have all been reported to differ between healthy and disease-associated clots (Pretorius, et al., 2011; Undas & Ariens, 2011; Weisel & Litvinov, 2013; Ząbczyk & Undas, 2017).

Among the available imaging techniques, scanning electron microscopy (SEM) remains one of the most widely used methods for quantitative analysis of fibrin structure because it provides sufficient resolution to visualize individual fibrin fibers. SEM has been extensively applied to whole blood clots (Pretorius, et al., 2011; Undas & Ariens, 2011; Weisel & Litvinov, 2013; Ząbczyk & Undas, 2017; Risman, et al., 2022), platelet-poor plasma clots (Risman, et al., 2024; Cai, et al., 2026*a*; Cai, et al., 2026*b*), and purified fibrinogen clots (Risman, et al., 2024; Cai, et al., 2026*b*) to quantify fibrin fiber diameter, fiber length, branching, and other structural parameters. In particular, fibrin fiber diameter is one of the most frequently reported measurements and is commonly used to compare clot structure across disease states, experimental conditions, and biochemical compositions (Mills, et al., 2002; Undas, et al., 2009; Daraei, et al., 2021; Cai, et al., 2026*a*).

Despite its widespread use, conventional SEM preparation of fibrin clots is labor intensive and time consuming. Typical protocols involve fixation, washing, dehydration, drying, mounting, conductive sputter coating, and imaging. Conductive coating is commonly required to prevent charging of biological samples during SEM imaging; however, the deposited metal layer introduces an additional nanoscale coating onto the fiber surface and may influence quantitative measurements of fiber diameter. This issue is particularly relevant when analyzing fibrin fibers, whose diameters often range from only tens to hundreds of nanometers (Belcher, et al., 2023; Risman, et al., 2024; Cai, et al., 2026*a*; Cai, et al., 2026*b*).

In addition, many SEM preparation procedures currently used for purified fibrinogen clots are adapted from protocols originally developed for plasma clots (Pieters, et al., 2008; Risman, et al., 2024). Because purified fibrinogen systems lack many plasma components, some of these preparation steps may be unnecessary and may increase preparation time without improving measurement quality. Therefore, there is a need for simplified SEM workflows that reduce sample processing while maintaining reliable quantitative measurements.

Indium tin oxide (ITO) is an inexpensive, flat, conductive substrate that may provide an alternative approach for SEM imaging by reducing or eliminating the need for conductive sputter coating (Liv, et al., 2013). In this study, we evaluate ITO substrates for SEM imaging of fibrin fibers, investigate the effects of sputter coating duration on measured fibrin fiber diameter, and develop a simplified preparation protocol for purified fibrinogen clots. We further compare this simplified ITO-based workflow with a previously standardized SEM protocol (Cai, et al., 2026*a*) to determine whether reliable quantitative measurements can be obtained with fewer preparation steps.

## Materials and Methods

### Reagents

Platelet-poor, pooled normal human plasma was purchased from George King Bio-Medical (Product No. 0010; lot 7716). The manufacturer-reported fibrinogen concentration, determined using the functional Clauss method, was 3.2 mg/mL. Peak 1 purified human fibrinogen was purchased from Enzyme Research Laboratories (Cat. No. P1 FIB; lot P1 Fib 5980A). The manufacturer-reported protein concentration was 27.57 mg/mL, as determined by absorbance at 280 nm, and the material was reported to be 100% clottable based on an in-house clottability assay. Human α-thrombin was obtained from Enzyme Research Laboratories (Cat. No. HT1002a; lot-specific activity, 3044 NIH U/mg). Plasma, fibrinogen, and thrombin stocks were divided into single-use aliquots and stored at −80°C. Before each experiment, aliquots were thawed at 37°C and used immediately.

### Clot Formation Surfaces and SEM Imaging Substrates

ITO-coated glass was used as a conductive substrate for both clot formation and SEM imaging. ITO substrates were unpatterned ITO-coated glass microscope slides (25 × 75 mm; Ossila, Sheffield, UK), which were scored and cut with a diamond scribe into six individual pieces before use. In the standardized lid-to-carbon-tape method, clots were formed in microtube lids following the previously described SEM preparation protocol (Cai, et al., 2026*a*), then removed from the lids and mounted onto carbon tape for SEM imaging.

### Fibrin Clot Formation

Plasma and purified fibrinogen clots were prepared at final fibrinogen and thrombin concentrations of 2.9 mg/mL and 1.0 NIH U/mL, respectively. Clot formation was initiated by combining the fibrinogen-containing solution with thrombin and CaCl_2_ in Tris-buffered saline (50 mM Tris, 100 mM NaCl, pH 7.4). Plasma clots were prepared with 20 mM CaCl_2_ to recalcify the citrate-anticoagulated plasma. Purified fibrinogen clots were prepared with 9.5 mM CaCl_2_ because no citrate was present in the purified system. All clots were polymerized for 2 h at room temperature before fixation.

### Experimental Design

The study consisted of four experimental comparisons designed to evaluate sputter-coating effects, the use of ITO-coated glass as a conductive substrate for clot formation and subsequent SEM imaging, and the feasibility of simplifying the purified fibrinogen clot preparation workflow. An overview of the experimental design is provided in Table 1.

**Table 1.** Experimental Design and Study Comparisons.

Comparison	Sample	Workflow	Coating	Purpose
Coating time on ITO	Purified fibrinogen	ITO + Simplified protocol	0, 45, 90 s	Test simplified ITO method with different coating times
Coating time on ITO	Plasma	ITO + Standard wash/dehydration steps	0, 45, 90 s	Test coating effect on ITO
Protocol comparison	Purified fibrinogen	ITO + Simplified protocol versus Standardized lid-to-carbon-tape	Matched	Test whether simplified ITO protocol gives comparable fiber diameters
ITO versus standardized lid-to-carbon-tape	Plasma	ITO + Standard wash/dehydration steps versus Standardized lid-to-carbon-tape	Matched	Test whether ITO changes measured fiber diameter

### ITO-based Simplified Preparation of Purified Fibrinogen Clots

For the simplified ITO protocol, 10 µL of the purified fibrinogen clotting mixture was pipetted directly onto an ITO-coated glass substrate placed in a 12-well plate. After polymerization for 2 h at room temperature, the clot was fixed on the substrate by complete immersion in 2 mL of 2% glutaraldehyde prepared in sodium cacodylate buffer (50 mM sodium cacodylate, 100 mM NaCl, pH 7.4) overnight. Samples were covered during polymerization and fixation to minimize evaporation. After fixation, the fixation buffer was carefully removed by pipette, and 2 mL of 100% ethanol was added to cover the clot on the ITO substrate. The ITO samples were then transferred with tweezers to a Tousimis Samdri-PVT-3D critical point dryer for critical point drying.

This simplified workflow (Fig. 1) was designed to reduce sample handling by omitting the repeated buffer washing steps and the graded ethanol dehydration series used in the previously standardized fibrin SEM preparation protocol (Cai, et al., 2026*a*).

**Fig. 1.**
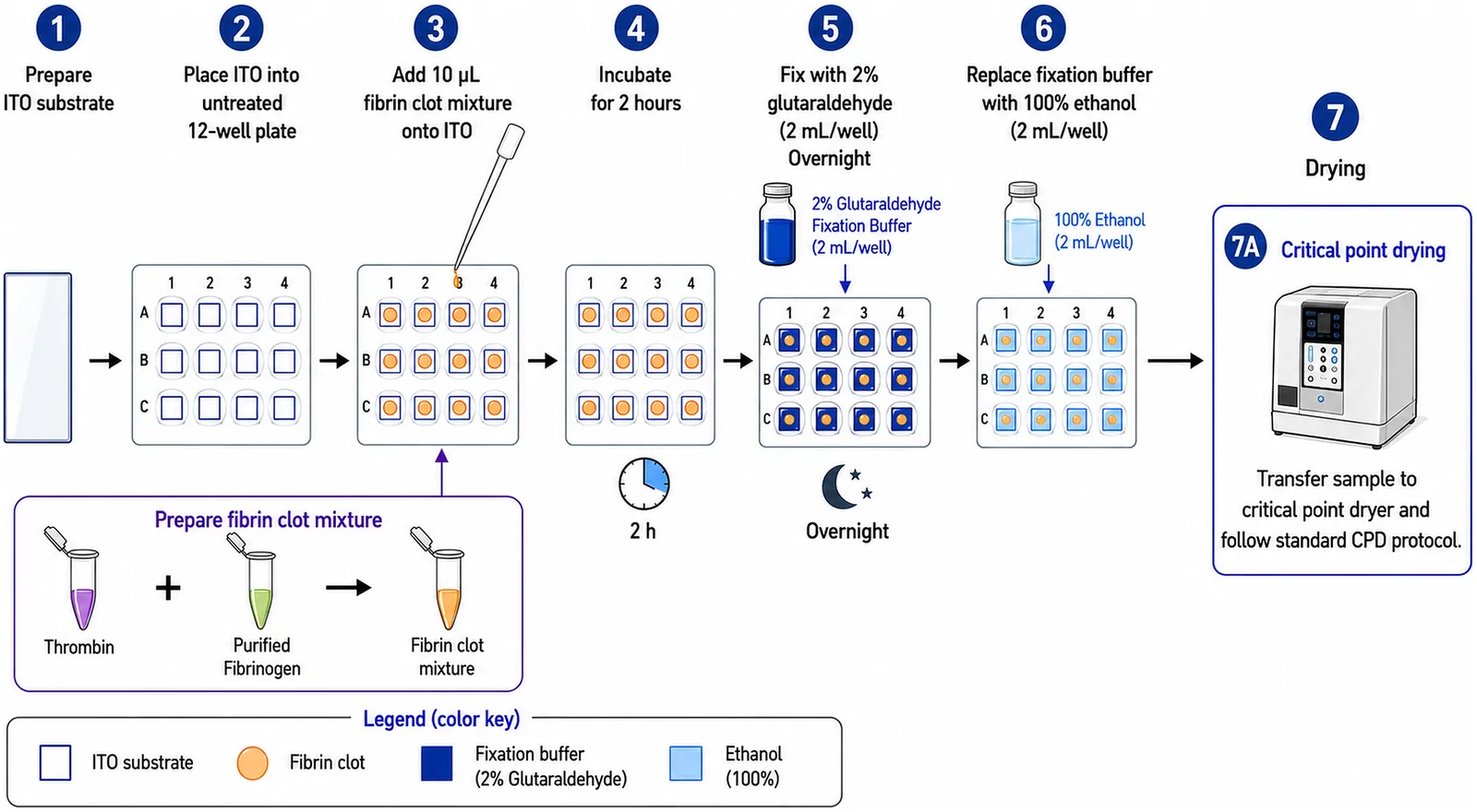
Simplified purified fibrinogen sample preparation for SEM. (This schematic was created with assistance from ChatGPT).

### Preparation of Plasma Clots on ITO

Plasma clots were prepared directly on ITO substrates to evaluate whether conductive ITO could support SEM imaging of plasma-derived fibrin networks with reduced or no sputter coating. Because plasma clots contain additional plasma proteins, we included wash steps to remove non-fibrin components and leave primarily the fibrin fiber network. Plasma samples were therefore processed using a similar, previously described conservative standardized preparation workflow, except that clot formation was performed on ITO instead of in the microtube lid (Cai, et al., 2026*a*). After polymerization, samples were rinsed with sodium cacodylate buffer, fixed in 2% glutaraldehyde, rinsed again, dehydrated through increasing ethanol concentrations, and dried by critical point drying. Dried samples were either left uncoated or sputter-coated with gold for 45 or 90 s.

### Comparison With the Standardized Lid-to-carbon-tape Protocol

To determine whether the simplified ITO method produced fiber diameter measurements comparable to the previously standardized SEM method, purified clots were prepared using two workflows. In the first workflow, clots were formed directly on ITO substrates and processed using the simplified ITO method described above. In the second workflow, clots were formed in microtube lids (standardized lid-to-carbon-tape) and processed using the previously standardized wash, fixation, dehydration, drying, and sputter-coating procedure. For direct comparison, ITO and standardized lid-to-carbon-tape samples were sputter-coated for the same duration before SEM imaging. This design allowed the effect of the simplified ITO-based preparation to be evaluated without introducing differences in coating time.

### Sputter Coating Conditions

Dried samples were imaged either without sputter coating or after gold sputter coating using a Cressington Model 108 sputter coater. For coating-time experiments, samples were assigned to 0, 45, or 90 s coating conditions. For direct comparisons between the simplified ITO method and the standardized lid-to-carbon-tape method, both sample groups were coated for the same duration.

### SEM Imaging

Samples were imaged using a Zeiss Gemini 300 scanning electron microscope under high vacuum at 30,000× magnification. ITO-coated glass substrates were mounted directly onto aluminum SEM stubs using double-sided conductive carbon tape. For samples prepared in microcentrifuge tube lids, the dried clots were carefully removed with forceps and transferred onto double-sided conductive carbon tape attached to aluminum SEM stubs. For each experimental condition, two independently prepared clots were imaged. Eight non-overlapping fields of view were collected from each clot, yielding 16 SEM images per condition. Images were acquired from widely separated regions of the clot while avoiding the film-like edge region and obvious drying or handling artifacts.

For uncoated samples prepared on ITO substrates, imaging was performed at low accelerating voltage (≤5 kV) using the InLens secondary electron detector to minimize charging and enhance surface contrast. To further reduce charging and beam-induced artifacts, prolonged scanning of the same area was avoided (typically <1 min per field of view), and regions showing obvious charging artifacts were excluded from analysis.

### Fiber Diameter Analysis

Fibrin fiber diameters were measured from SEM micrographs using the same analysis procedure across all experimental groups, as previously described (Cai, et al., 2026*a*). Measurements were performed using the freely available NIH ImageJ software. A grid was overlaid on each image, and at least 70 fibers were measured per image. At each grid location, the fiber closest to the grid point was selected, with care taken to avoid measuring the same fiber more than once. Only clearly resolved fibers in the visible upper layer of the network were measured to minimize ambiguity caused by overlapping fibers. For each image, the median, lower-quartile, and upper-quartile fiber diameters were calculated and used for statistical comparisons. Median fiber diameters are presented in Figures 2 and 3, while lower- and upper-quartile fiber diameters are presented in Supplementary Figures 3 and 4, respectively.

**Fig. 2.**
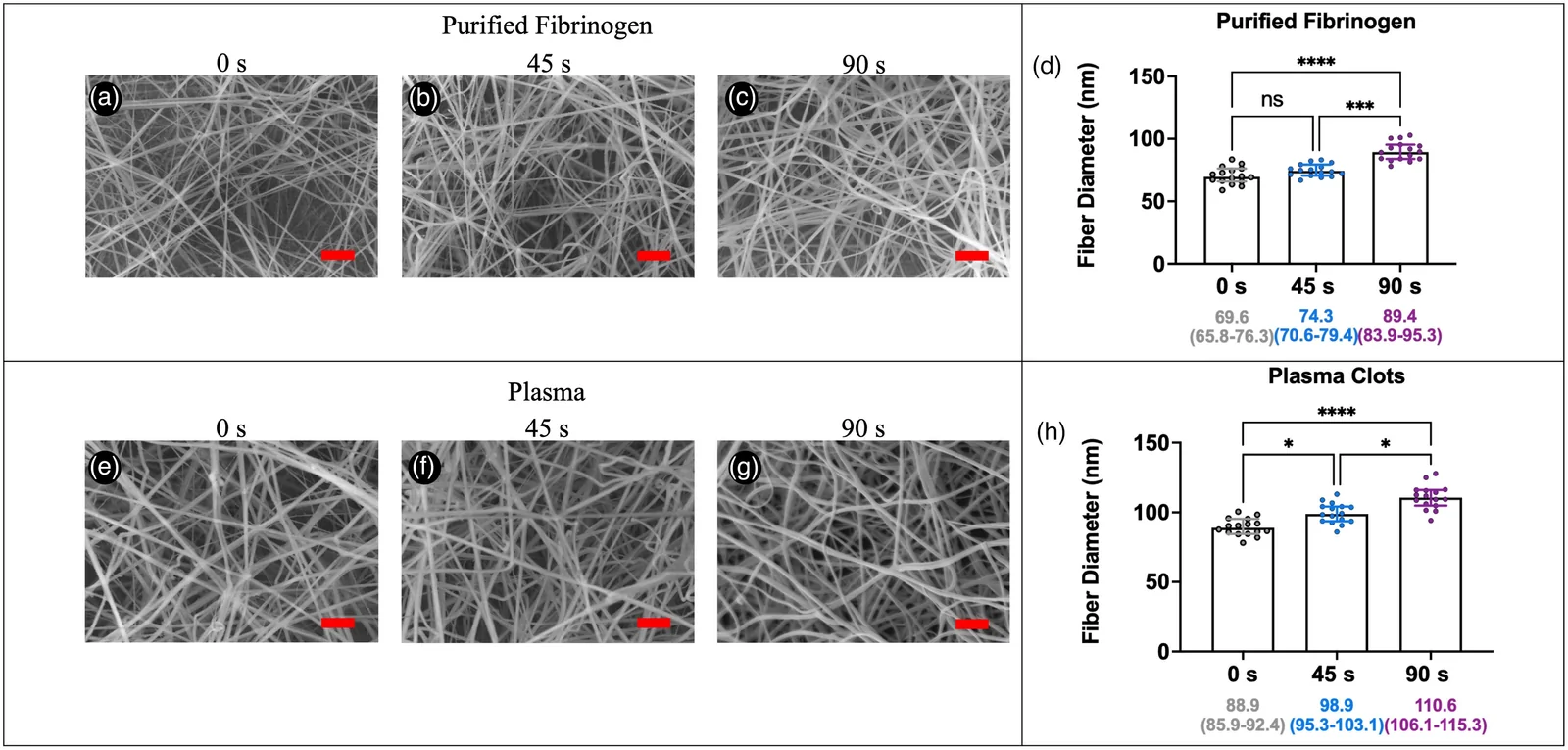
Coating-time dependence of fibrin fiber diameter measurements on ITO. Scale bar, 1 µm. (**a–c**) Purified fibrinogen clots (simplified ITO protocol, Fig. 1) with coating times of 0, 45, and 90 s. (**d**) Image-level medians of fiber diameter (*n* = 16 SEM images per timepoint); points are per-image medians; line = median across images; bars = 95% CI. (**e–g**) Plasma clots with coating times of 0, 45, and 90 s. (**h**) Image-level medians of fiber diameter (*n* = 16 per timepoint); points are per-image medians; line = median across images; bars = 95% CI. Statistical significance was assessed using the Kruskal–Wallis test followed by Dunn's multiple-comparisons test. Asterisks indicate adjusted *p* values: ns, *p* > 0.05; *, *p* ≤ 0.05; **, *p* ≤ 0.01; ***, *p* ≤ 0.001; and ****, *p* ≤ 0.0001.

**Fig. 3.**
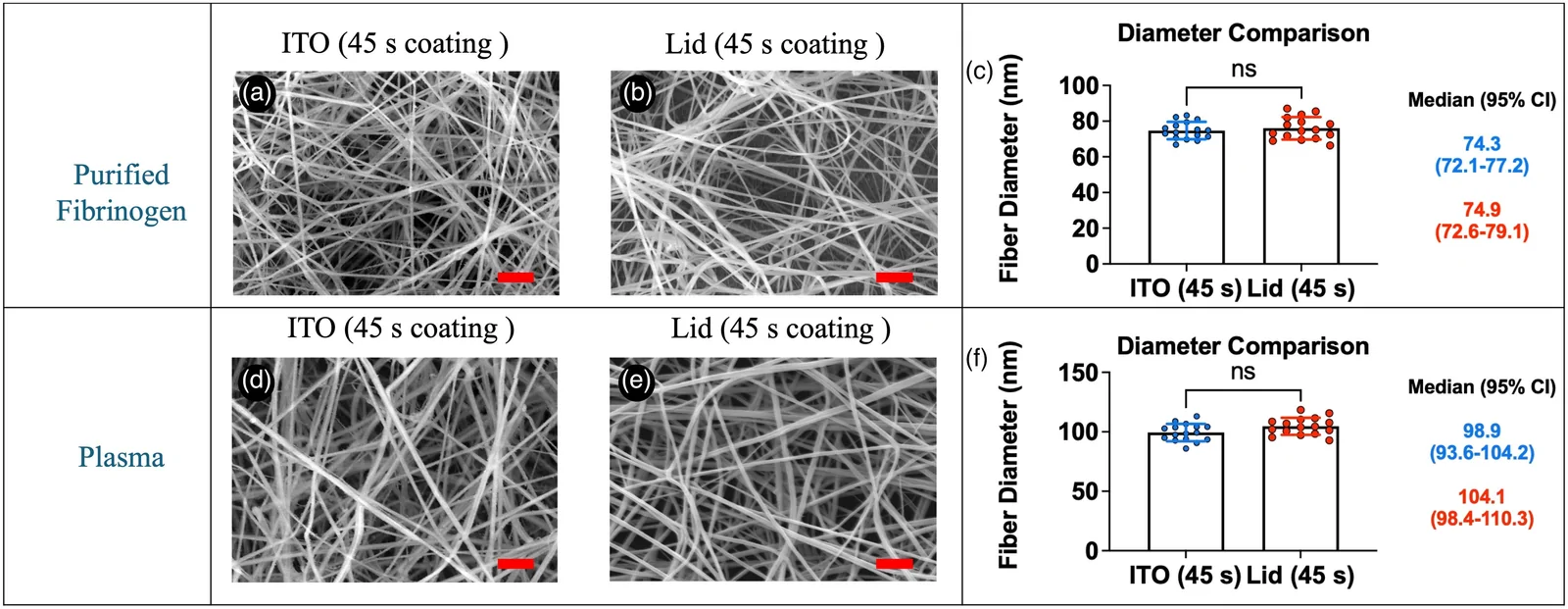
Validation of the simplified ITO protocol and comparison with the standardized lid-to-carbon-tape workflow. Scale bar, 1 µm. (**a**) Purified fibrinogen clot formed on ITO using the simplified protocol (45 s coating). (**b**) Purified fibrinogen clot formed in a microtube lid using the standardized lid-to-carbon-tape protocol (45 s coating). (**c**) Fiber diameter comparison for (**a**) versus (**b**): Each point is the median diameter from one SEM image (*n* = 16 images/condition); line = median across images; bars = 95% CI. (**d**) Plasma clot formed on ITO (45 s coating). (**e**) Plasma clot formed in a microtube lid (45 s coating). (**f**) Fiber diameter comparison for (**d**) versus (**e**): Each point is the median diameter from one SEM image (*n* = 16 images/condition); line = median across images; bars = 95% CI. Statistical significance was assessed using a two-tailed Mann–Whitney U test. ns, not significant (*p* > 0.05).

### Statistical Analysis

For each SEM image, the median, lower-quartile, and upper-quartile fiber diameters were calculated from the individual fiber measurements. Each SEM image was treated as one independent experimental unit, resulting in *n* = 16 image-level values per condition for each summary measure. Statistical analyses were performed separately for the image-level median, lower-quartile, and upper-quartile fiber diameters.

For comparisons among the three sputter-coating times (0, 45, and 90 s), differences were assessed using the Kruskal–Wallis test followed by Dunn's multiple-comparisons test with adjustment for multiple pairwise comparisons. These analyses were conducted separately for purified fibrinogen clots prepared using the simplified ITO protocol and for plasma clots prepared on ITO.

For comparisons between the ITO-based workflow and the standardized lid-to-carbon-tape workflow at a coating time of 45 s, differences were assessed using a two-tailed Mann–Whitney U test. Purified fibrinogen and plasma clots were analyzed separately.

The image-level median fiber-diameter results are presented in Figures 2 and 3, whereas the corresponding lower- and upper-quartile analyses are presented in Supplementary Figures 3 and 4, respectively. Adjusted *p* values are reported for Dunn's multiple-comparisons tests. Statistical significance was defined as *p* ≤ 0.05. Statistical analyses were performed using GraphPad Prism [Prism 10, GraphPad Software, Boston, MA, USA].

## Results

### Coating Time on ITO (Purified Fibrinogen; Simplified ITO Protocol)

Sputter coating time produced a monotonic increase in the measured fibrin fiber diameter for purified fibrinogen clots prepared on ITO using the simplified protocol (Fig. 2). Across 16 SEM images per condition, the median image-level diameter increased from 69.6 nm (0 s) to 74.3 nm (45 s) and 89.4 nm (90 s). The coating-time effect was significant by Kruskal–Wallis (*H* = 30.40, *p* < 0.0001; *n* = 16 images/group), and Dunn's multiple-comparisons test showed 0 versus 45 s, *p* = 0.58; 0 versus 90 s, *p* < 0.0001; 45 versus 90 s, *p* = 0.0002.

### Coating Time on ITO (Plasma; Standard Protocol on ITO)

Plasma clots prepared on ITO showed a similar coating-dependent increase in measured fiber diameter (Fig. 2). The median image-level diameter increased from 88.9 nm (0 s) to 98.9 nm (45 s) and 110.6 nm (90 s) across 16 SEM images per condition. Kruskal–Wallis indicated significant differences among coating times (*H* = 29.34, *p* < 0.0001; *n* = 16 images/group), and Dunn's test yielded 0 versus 45 s, *p* = 0.02; 0 versus 90 s, *p* < 0.0001; 45 versus 90 s, *p* = 0.02.

### Simplified ITO Preparation versus Standardized Lid-to-carbon-tape (Purified Fibrinogen, 45 s Coating)

The simplified ITO preparation for purified fibrinogen produced fiber diameter measurements comparable to the standardized lid-to-carbon-tape workflow (Fig. 3). Across 16 SEM images per condition (each point representing the median fiber diameter within one image), the median image-level diameter was 74.3 nm for ITO (45 s) and 74.9 nm for standardized lid-to-carbon-tape (45 s). A two-tailed Mann–Whitney U test showed no significant difference between the two workflows (*U* = 116, *p* = 0.67).

### ITO-based Workflow versus Standardized Lid-to-carbon-tape Workflow in Plasma Clots (45 s Coating)

For plasma clots processed with the standardized workflow, measured fiber diameters on ITO were not significantly different from those formed on microtube lids (Fig. 3). The median image-level diameter across 16 SEM images per condition was 98.9 nm on ITO (45 s) and 104.1 nm on standardized lid-to-carbon-tape (45 s). This difference was not statistically significant by a two-tailed Mann–Whitney U test (*U* = 79, *p* = 0.07).

## Discussion

This study addressed four practical questions relevant to reproducible SEM measurement of fibrin fiber diameter: (i) how sputter-coating time affects measured diameters on ITO, (ii) whether an ITO-based simplified workflow is feasible for purified fibrinogen clots, (iii) whether using ITO as the clot formation and imaging surface biases plasma fiber diameter measurements relative to the standardized lid-to-carbon-tape workflow, when the remaining preparation steps are held constant, and (iv) whether the simplified ITO workflow yields comparable results to the standardized lid-based workflow for purified fibrinogen.

Across both purified fibrinogen and plasma clots formed on ITO, sputter coating time produced a monotonic increase in the measured fiber diameter. For purified fibrinogen (simplified ITO protocol), the median of image-level medians increased from 69.6 nm (0 s) to 74.3 nm (45 s) and 89.4 nm (90 s), with significant differences driven primarily by the 90 s condition. Plasma clots formed on ITO showed a similar shift from 88.9 nm (0 s) to 98.9 nm (45 s) and 110.6 nm (90 s) Analyses of the image-level lower and upper quartiles led to the same statistical conclusions as the median-based analysis (Supplementary Figs. S3, S4). These results reinforce that sputter coating is not a neutral imaging step: increasing coating time can introduce a systematic, directionally consistent bias in apparent fibrin fiber diameter. The magnitude of the shift is consistent with the expected addition of a nanoscale conductive gold layer during sputter coating; however, the exact coating thickness was not directly measured in this study and can vary with sputtering current, chamber pressure, working distance, and sample geometry. Thus, the observed diameter increases likely reflect, at least in part, the added coating layer rather than underlying structural changes in the fibrin network. Practically, this argues that coating conditions must be tightly standardized and explicitly reported whenever fiber diameter is used as a quantitative readout across experiments or laboratories.

A central motivation of using ITO is that its conductive surface can reduce charging artifacts and enable imaging with reduced or even no sputter coating, which would eliminate one major source of measurement variability. In our SEM workflow, uncoated imaging on ITO was performed at low accelerating voltage (≤5 kV) using the InLens detector, and with limited dwell time per field of view to minimize charging and beam-induced artifacts. However, conductivity of the substrate does not guarantee charge dissipation for all sample geometries. If portions of the clot are elevated or poorly attached relative to the conductive ITO surface, such as thick deposits, folded regions, or detached material, those regions may still charge during SEM imaging. We therefore used a small reaction volume (10 µL) to form shallow dome-shaped clots on the ITO surface and selected imaging regions that appeared well-coupled to the conductive substrate. Assuming a sample area of 25 mm^2^, the average clot height is 40 µm.

The simplified ITO-based preparation for purified fibrinogen produced measurements comparable to the standardized lid-based workflow at matched coating time (45 s). The median image-level fiber diameter was 74.3 nm on ITO versus 74.9 nm on standardized lid-to-carbon-tape (*n* = 16 images per condition; Mann–Whitney U test, *p* = 0.6689), indicating no detectable bias introduced by the simplified ITO-based workflow in the purified system. The corresponding analyses of the image-level lower and upper quartiles also showed no significant difference between the two preparation workflows (Supplementary Figs. S3, S4). This supports the use of the simplified ITO protocol as a practical alternative for purified fibrinogen clots, reducing handling steps while preserving quantitative readouts.

In contrast, plasma clots contain additional plasma proteins and non-fibrin components that can remain associated with the network after polymerization. Proteomic studies illustrate the extent of this complexity. Talens et al. identified 18 non-covalently clot-bound proteins after extensive washing, including fibronectin, plasminogen, factor XIII, α2-macroglobulin, α1-antitrypsin, and several apolipoproteins (Talens, et al., 2012). More recently, high-throughput analyses have quantified up to 476 proteins in washed plasma fibrin clots, including coagulation and fibrinolytic proteins such as factor XIII, plasminogen, prothrombin, antithrombin, and α2-antiplasmin; adhesive proteins such as fibronectin and von Willebrand factor; and apolipoproteins, complement components, and inflammatory proteins (Bryk, et al., 2020). The abundance of several clot-associated proteins has also been associated with clot permeability and network density.

Residual non-fibrin material in minimally washed plasma samples is therefore likely to comprise a heterogeneous mixture of retained plasma proteins and lipoprotein-associated components, together with material indirectly associated with or physically entrapped within the fibrin network. Because the deposits observed by SEM were not compositionally analyzed in this study, their precise molecular identity cannot be determined from morphology alone. As a result, conservative wash steps remain important if the goal is to measure fibrin fiber diameters comparably to established standardized workflows. Consistent with this, we retained the washing and graded dehydration steps for plasma samples in the standard protocol, as illustrated in the Supplement, to reduce the risk of residual material obscuring fibers or confounding fiber-diameter measurements.

Importantly, when plasma samples were processed with the same standardized workflow, the choice of ITO-based workflow versus standardized lid-to-carbon-tape workflow did not significantly alter measured diameters at 45 s coating (98.87 nm on ITO versus 104.1 nm on standardized lid-to-carbon-tape; *n* = 16 images per condition; Mann–Whitney U test, *p* = 0.0671). These findings suggest that, under a controlled protocol, the use of ITO as the clot formation and imaging surface is unlikely to be a dominant driver of between-study variation in plasma fiber diameter, and that protocol differences (including coating conditions and preparation steps) are more consequential.

Although residual non-fibrin material is undesirable when the goal is standardized fibrin fiber diameter measurement, its retention may be informative in a different experimental context. The accumulation of material on or around fibrin fibers in minimally washed plasma samples suggests that reduced-washing workflows may help preserve plasma components associated with or entrapped within the fibrin network that are otherwise removed during conventional preparation. Determining the identity and mode of association of these components would require targeted proteomic, immunolabeling, or correlative imaging approaches. Thus, while conservative washing remains preferable for quantitative diameter measurements in plasma, simplified or minimally washed ITO-based preparation may provide a useful platform for studying interactions between fibrin fibers and associated proteins or other soluble components in targeted experiments. In purified fibrinogen systems, where fewer non-fibrin components are present, the simplified ITO workflow may be especially useful for controlled studies in which defined proteins are added to fibrinogen before polymerization. Because the clot can be formed and imaged directly on the conductive ITO surface with fewer washing and transfer steps, this approach may help preserve protein–fibrin associations and reduce preparation-induced loss or disruption of loosely associated material.

Together, these findings highlight two sources of variability that can plausibly contribute to the wide spread of “normal” fiber diameters reported across laboratories: differences in sputter-coating conditions and differences in preparation workflows (particularly for complex samples such as plasma). Using conductive ITO substrates can reduce charging constraints and enable protocol simplification in purified systems, but plasma samples still benefit from conservative washing and dehydration steps to isolate fibrin fibers for consistent quantification.

For matching fibrinogen and added thrombin concentrations (2.9 mg/mL and 1 NIH U/mL, respectively), plasma clots consistently showed a larger fiber diameter compared to clots formed from purified fibrinogen. This observation has been made before (Cai, et al., 2026*b*), and there may be multiple reasons for this finding. First, the effective thrombin activity in plasma might be reduced, due to thrombin inhibition (e.g., by antithrombin). Lowered thrombin activity would result in thinner fibers (Cai, et al., 2026*b*). Second, plasma proteins and other molecules might get incorporated into fibrin fibers and thereby thicken fibers. The unwashed plasma fibrin fibers (Supplementary Figs. S1, S2) strongly suggest that plasma proteins and molecules associate with fibrin fibers. In line with this argument are mass spectrometry studies showing that numerous plasma proteins and molecules are bound to fibrin fibers (Talens, et al., 2012; Bryk, et al., 2020).

## Conclusions

Using ITO-coated glass as a conductive substrate enables a simplified SEM preparation workflow for purified fibrinogen clots without altering measured fiber diameter relative to the standardized lid-based method at matched coating time. For plasma clots, the ITO-based clot formation and imaging workflow did not significantly change measured fiber diameters compared with the standardized lid-to-carbon-tape workflow when the remaining preparation steps were matched. However, washing and graded dehydration steps remain important for plasma samples because additional plasma proteins may obscure fibrin fibers or confound diameter measurements.

Sputter-coating time systematically increases the measured fiber diameter on ITO for both purified fibrinogen and plasma, consistent with the expected deposition thickness per unit time. Therefore, coating conditions should be standardized and explicitly reported to support meaningful comparisons across experiments and laboratories. The simplified ITO workflow may also be useful for future controlled studies of fibrin interactions with defined added proteins or associated components, because reduced washing and transfer steps may help preserve loosely associated material. Finally, while ITO can mitigate charging and facilitate reduced-coating imaging at low accelerating voltage, reliable uncoated imaging depends on maintaining close coupling between the sample and the conductive surface, motivating thin deposits and careful selection of imaging regions.

## Availability of Data and Materials

The authors have declared that no datasets apply for this piece.

## Supplementary Material

ozag116_Supplementary_Data

## Supplementary Material

To view Supplementary material for this article, please visit https://doi.org/10.1093/mam/ozag116.
