# Supplementary material for: Indium Tin Oxide Substrates Enable Coating-Free SEM Imaging and Simplified Preparation of Purified Fibrinogen Clots
Source: Microsc Microanal. 2026 Nov 11;32(6):ozag116. doi: 10.1093/mam/ozag116 (PMC13752228; doi:10.1093/mam/ozag116)
Supplement: Supplementary file 1 [file ozag116_supplementary_data.docx]

**Supplement**

**Indium Tin Oxide (ITO) Substrates Enable Coating-Free SEM Imaging and Simplified Preparation of Purified Fibrinogen Clots**

Can Cai^1^, Caela Flake^1^, Arezoo Nameny^1^, Nathan E. Hudson^2^, Brittany E. Bannish^3^, Martin Guthold^1†^

^1^ Department of Physics, Wake Forest University, Winston-Salem, NC 27109

^2^ Department of Physics, East Carolina University, Greenville, NC 27885

^3^ Department of Mathematics and Statistics, University of Central Oklahoma, Edmond,

Oklahoma, 73034

^†^ Corresponding author: [gutholdm@wfu.edu](mailto:gutholdm@wfu.edu)

**Supplementary Figure 1. Plasma clot prepared using the simplified ITO workflow without washing and graded dehydration. Scale bar, 1 µm.** (A) Low-magnification SEM image showing the edge of a plasma clot prepared using the simplified ITO protocol. The sample contains abundant residual material associated with or deposited around the fibrin network, likely reflecting retained plasma components after omission of washing steps. (B) Higher-magnification SEM image showing that residual material obscures individual fibrin fibers, making the sample unsuitable for reliable fiber diameter quantification.

**Supplementary Figure 2. Zoomed-in image of plasma clot prepared using the simplified ITO workflow without washing and graded dehydration.** In a higher-resolution SEM image from a region with less residual material, individual fibrin fibers were more clearly visible, but additional material could still be observed attached to or deposited on the fiber surface.

**Supplementary Figure 3.** **Coating-time dependence (0, 45, 90 s) of fibrin fiber diameter measurements on ITO (Lower and upper quartiles).** (A) Lower quartiles of fiber diameter for purified fibrinogen clots. (B) Upper quartiles of fiber diameter for purified fibrinogen clots. (C) Lower quartiles of fiber diameter for plasma clots. (D) Upper quartiles of fiber diameter for plasma clots. For each condition, n = 16 SEM images per time point. Each point represents the corresponding lower or upper quartile of fiber diameter calculated from an individual SEM image. Horizontal lines indicate the median of the 16 image-level quartile values, and error bars represent the 95% confidence interval. Statistical significance was assessed using the Kruskal–Wallis test followed by Dunn’s multiple-comparisons test. Asterisks indicate adjusted p values: ns, p > 0.05; *, p ≤ 0.05; **, p ≤ 0.01; ***, p ≤ 0.001; and ****, p ≤ 0.0001.

**Supplementary Figure 4.** **Validation of the simplified ITO protocol and comparison with the standardized lid-to-carbon-tape workflow.** (A) Lower quartiles of fiber diameter for purified fibrinogen clots. (B) Upper quartiles of fiber diameter for purified fibrinogen clots. (C) Lower quartiles of fiber diameter for plasma clots. (D) Upper quartiles of fiber diameter for plasma clots. For each condition, n = 16 SEM images per time point. Each point represents the corresponding lower or upper quartile of fiber diameter calculated from an individual SEM image. Horizontal lines indicate the median of the 16 image-level quartile values, and error bars represent the 95% confidence interval. Statistical significance was assessed using a two-tailed Mann–Whitney U test. ns, not significant (p > 0.05).
